# Disparities in the prevalence of AIDS related opportunistic infections in Nigeria- implications for initiating prophylaxis based on absolute CD4 Count

**DOI:** 10.1186/1742-4690-9-S1-P147

**Published:** 2012-05-25

**Authors:** Chioma Onyinye Nwuba, Robert Okonkwo, Oluwafemi Abolarin, Ngozi Ogbu, Pauline Modebelu

**Affiliations:** 1 Pro-Act Msh, Ilorin, Nigeria

## Introduction

The differences that exist between the CD4 count strata at which certain opportunistic infections occur in sub Saharan Africa suggest that targeting prophylaxis at HIV patients in this part of the world based on data generated from developed countries may be one of the reasons why there is high HIV-related morbidity in Africa.

The aim of this study was to investigate the changing incidence of some AIDS defining illnesses in Northern and Southern parts of Nigeria, relating them to different CD4 count strata.

## Materials and methods

In this study, sputum samples of 234 HIV positive patients were analyzed for Mycobacterium tuberculosis and Pneumocystis jiroveci while 202 stool samples of HIV patients were analyzed for the presence of opportunistic intestinal parasites. We considered five CD4 strata (0 to 99, 100 to 199, 200 to 399, 400 to 499 and > 500 cells/µL). Incidence of the various opportunistic infections and their occurrence within each CD4 strata were estimated using simple statistical method. CD4 count of each patient was estimated using the Becton Dickenson FACSCount system.

## Results

The prevalence of Mycobacterium tuberculosis, Pneumocystis jirovecii, Cryptosporidium parvum, Isospora belli and Cyclospora spp were 10.3%, 41.9%, 30.8%, 24.2% and 4.4% respectively. All the opportunistic infections occurred at higher rates in CD4 counts less than 200cells/ul (p < 0.0001) except for tuberculosis which occurred highest at CD4 counts >200cells/ul (16 out of the 24 positive sputum smears were recovered in patients with CD4 counts >200 cells/ul). Despite pronounced immunosuppression, P. jiroveci was not detected in sputum samples of 8% patients with CD4 count <200 cells/ul.

Opportunistic parasites occurred almost exclusively at CD4 count <200 cells/ul. However, 6.4% of these parasites were isolated in patients with CD4 >200 cells/ul. Cryptosporidium parvum (30.8%) was the most frequently encountered opportunistic parasite, followed by Isospora belli (24.2%) and Cyclospora specie (4.4%).

## Conclusions

Although HIV related opportunistic infections are often reported to occur exclusively at CD4 count <200 cells/ul in patients; the result of this study shows that disparities exist and so, the possibility of opportunistic pathogens must remain in the differential diagnosis of infections in HIV patients independent of absolute CD4 count.

